# Superficial Myofibroblastoma in the Vulva Mimicking Aggressive Angiomyxoma: A Case Report and Review of the Literature

**DOI:** 10.1155/2019/1582714

**Published:** 2019-05-26

**Authors:** Wei-Xia Peng, Ryuichi Wada, Shoko Kure, Masaharu Fukunaga, Zenya Naito

**Affiliations:** ^1^Department of Integrated Diagnostic Pathology, Nippon Medical School, Japan; ^2^Department of Pathology, Misato Kenwa Hospital, Japan; ^3^Department of Pathology, Shinyurigaoka General Hospital, Japan

## Abstract

Background: Superficial myofibroblastoma (SMF) is a very rare benign mesenchymal tumor in the female lower genital tract. Only 46 cases have been reported in the English language literature, among which only 7 cases arose in the vulva. Sometimes SMF histologically mimics aggressive angiomyxoma (AA) in which massive myxoid change in stroma is characteristic. We herein report a case of vulvar SMF with prominent myxoid stroma and review the literature with the emphasis on the differential diagnosis of SMF and AA. Case presentation: a 37-year-old woman presented with a painless mass in the vulva. Magnetic resonance imaging (MRI) showed a well-circumscribed 7 cm mass in the subcutis of the vulva. The tumor was resected. Histopathologically, the tumor was characterized by sparsely populated spindle-shaped cells in the fibromyxoid stroma. Thin-walled blood vessels were detected. Mitoses or pleomorphism was not found. Tumor cells were positive for vimentin, ER, PgR, and desmin. Some cells were positive for alpha-SMA and CD34. All cells were negative for S100 protein. Conclusions: because SMF and AA show different clinical prognoses, distinguishing SMF from AA is important. However, SMF may share many common histological features with AA: superficial localization (above fascia), sharp borderline from adjacent tissue, expansive growth pattern; a specific vascular pattern will lead to an accurate diagnosis of SMF. Familiarization with the histological characteristics of the two entities will help to make a prognostic prediction.

## 1. Background

Superficial myofibroblastoma (SMF) is a very rare benign mesenchymal tumor of the female genital region. To date, only 46 cases have been reported in the English language literature [1–6]. Formerly, SMF was known as superficial cervicovaginal myofibroblastoma because it was believed to occur exclusively in the cervix and vagina. In 2005, Ganesan R et al. [2] proposed the term “superficial myofibroblastoma of the lower female genital tract” for this type of tumor because they found that some tumors with the same histological and immunohistochemical features also could occur in the vulva. Although some cases were reported thereafter, so far, only seven cases of SMF have been reported to arise in the vulva [2, 3, 6]. Thus, many gynecologists and pathologists might not be familiar with this type of tumor.

Histologically, SMF is characterized by myofibroblast proliferation in collagenous and myxoid stroma. The cellularity is always moderate to low. The collagen and myxoid stroma proportions may vary among cases. Blood vessels may be abundant in some cases. Sometimes, SMF may be associated with extensive edema in the stroma, which may make it difficult to distinguish it from aggressive angiomyxoma (AA). However, the clinical prognoses of the two tumors are different. Thus, familiarization with the characteristics of SMA and AA is important.

## 2. Case Presentation

**Figure 1:**
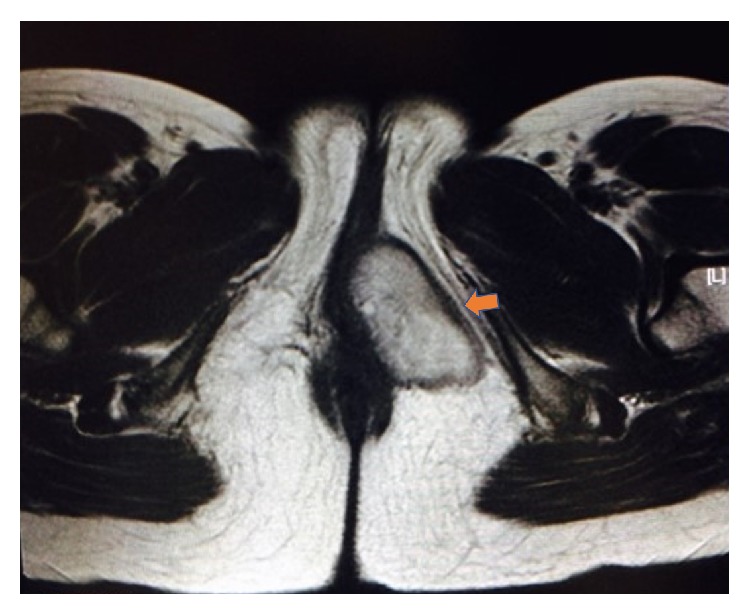
Magnetic resonance imaging of the pelvis showed a well-circumscribed mass in the vulvar subcutaneous region.

A 37-year-old woman, G1P1, was referred to our hospital due to an increase in size of a tumor in her vulva. The mass was first pointed out to her during her delivery one year earlier. The patient had no apparent symptoms. Magnetic resonance imaging (MRI) of the pelvis showed a well-circumscribed mass in the vulva (Figure 1). The patient underwent resection of the tumor, and the tumor was subjected to histological examination. There was no apparent evidence of recurrence one year after the resection.

Grossly, the tumor mass was located in the subcutis and measured 73×29 mm. There was no fibrous capsule, but the tumor was well circumscribed. The cut surface showed a yellowish-white mass with gelatinous change. No hemorrhage or necrosis was observed.

**Figure 2:**
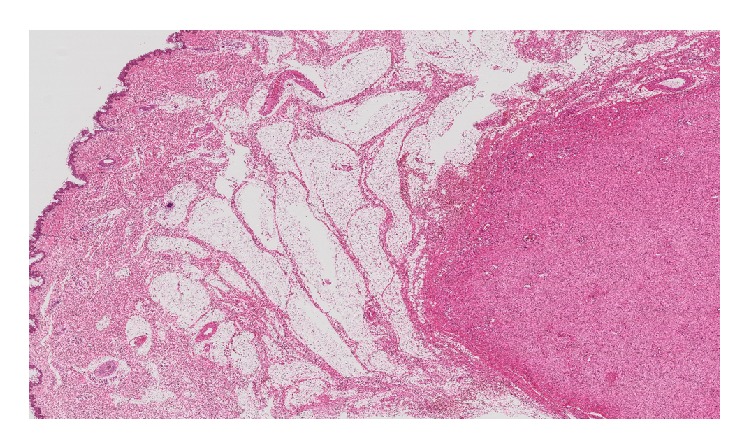
On histopathological examination (×10;HE), the tumor was located in the subcutaneous region. There is an uninvolved segment between the tumor and overlying squamous epithelium. The boundary between the tumor and adjacent tissue was well demarcated.

**Figure 3:**
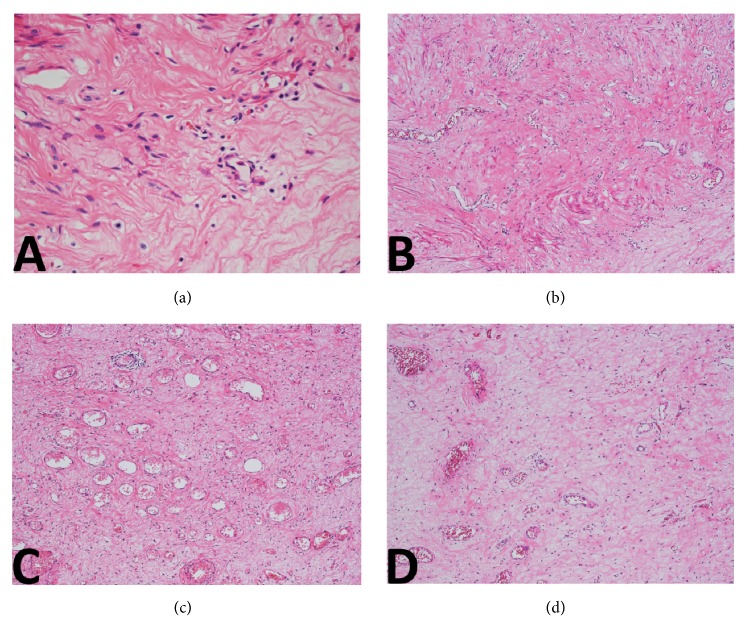
Tumor cells were short and spindle-shaped, arranged in no overt architecture. The nuclei were oval, without prominent atypia. The intervening matrix was edematous and myxoid ((a); ×400;HE). In some regions, fine collagen as well as dense collagen was detected ((b); ×100;HE). The vast majority of blood vessels were small and thin-walled ((c); ×100;HE). Some medium-sized blood vessels were also identified within the lesion ((d); ×100;HE). There was no specific distribution pattern of the vascularity.

**Figure 4:**
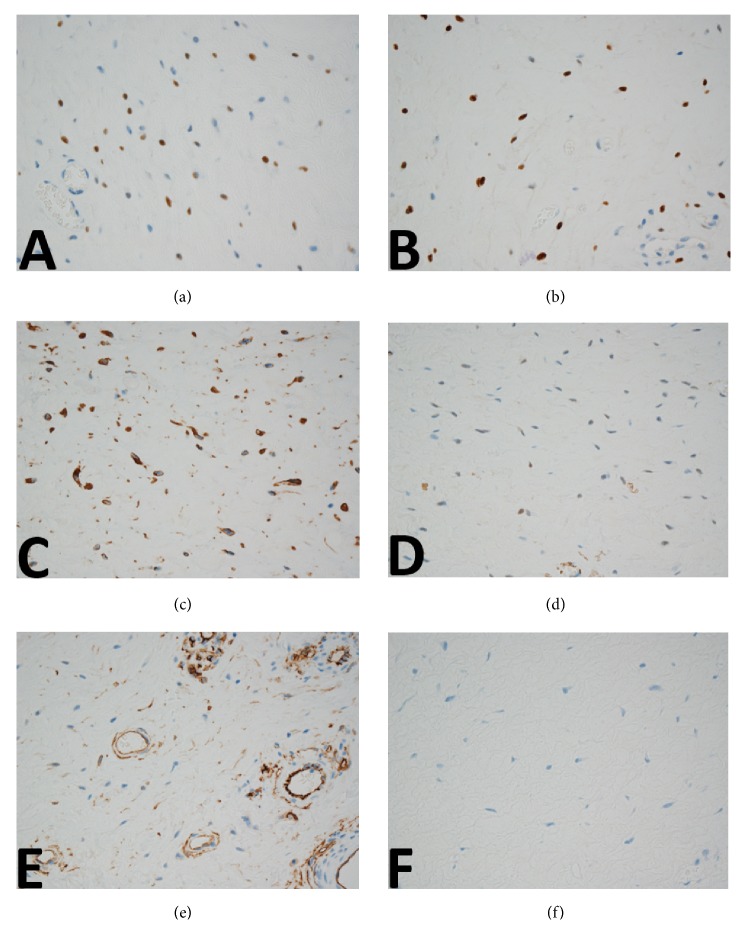
Immunohistochemically, positive nuclear staining for ER (a) and PgR(b) was observed. Tumor cells showed cytoplastic positivity for desmin (c) and *α*SMA (d). CD34 (e) and S100 protein were negative in all cells(f).

On histopathological examination (Figure 2), the boundary between tumor and adjacent tissue was clear. Tumor cells were short and spindle-shaped without prominent atypia, arranged in no overt architecture. No necrosis or mitoses were identified. The stroma was edematous and myxoid; fine collagen as well as dense collagen was detected in some regions. The vast majority of blood vessels were small-sized with thin walls. Some medium-sized blood vessels were also identified within the lesion (Figure 3). There was no specific distribution pattern of the vascularity. Immunohistochemical studies were performed using the primary antibodies listed in Table 1. On immunohistochemical analysis, most tumor cells showed positivity for vimentin, ER, PgR, and desmin. Some tumor cells showed positive for alpha-SMA and CD34. The tumor cells were uniformly negative for S100 protein (Figure 4). The Ki-67 labeling index was less than 2%.

## 3. Discussion and Conclusions

Diagnosing genital mesenchymal tumors is usually challenging because they are rare, and some of them show many similar clinicopathologic features. Hypocellularity, marked edema, and myxoid change, as well as prominent blood vessel proliferation in the stroma, made the present case atypical and difficult to distinguish from AA. SMF, as well as many other genital mesenchymal tumors, may exhibit massive myxoid change in the stroma. Especially in tumors in superficial regions, edema and myxoid change due to external stimulation is a common secondary change. Therefore, a myxoid stroma is not an exclusive finding of AA. Although in rare cases, SMF may show marked proliferation of blood vessels in the stroma, the blood vessels are always small- to medium-sized. Large-size vessels with thick muscular walls or hyalinized change are seldom seen. Furthermore, the vessels have a trend to concentrate in the central region of the lesion. However, blood vessels in AA are always abundant and multifarious, varying from capillary-like to large-caliber vessels with thick muscular walls. Moreover, an arborizing vascular pattern, as seen in SMF and some other mesenchymal tumors, is always absent. Immunostaining also provides little help to the discrimination because both SMF and AA, as well as many other mesenchymal tumors in the female genital tract, may show positive for ER, PgR, desmin, alpha-SMA, and CD34 [1–3] (Table 2).

One key point of difference between SMF and AA is the location of the mass. SMF, as its name implies, presents as a mass in the superficial region, whereas AA is characterized by its deep location. Although there is no histological definition of “superficial,” based on a radiological report, a location can be termed superficial if it is located in the regions above the muscle [7]. Accordingly, SMF can be described as a mass located above the fascia. Another distinguishing point between SMF and AA is the growth pattern of the tumor. SMF always shows an expansive growth pattern with clear margins, whereas AA shows an aggressive growth pattern and infiltration to the surrounding tissues. Thus, some entrapped tissues, such as neuron fibers, fat tissue, large-size blood vessels, and muscles, can be observed within the AA. Identifying these entrapped tissues is useful in leading to an accurate diagnosis. Because of the infiltrating growth pattern, complete resection is difficult and often results in clinical recurrence.

In addition to AA, other potential mimics of SMF include mammary type myofibroblastoma, angiomyofibroblastoma, and cellular angiofibroma, which all may show similar histological findings with SMF (Table 2). On the other hand, when a tumor shows prominent myxoid changes, a myxoid type dermatofibrosarcoma protuberance should be considered. AMF differs in that the cells usually show an epithelioid appearance, and the cells are often arranged around blood vessels, which we did not identify in our case.

In summary, histologically, SMF may mimic AA by showing massive myxoid and edema changes in some cases. Because SMF and AA have different clinical prognoses, distinguishing SMF from AA is important. Superficial location, sharp borderline from adjacent tissue, expansive growth pattern, and specific vascular pattern may all help to make an accurate diagnosis.

## Figures and Tables

**Table 1:** Primary antibodies used in this study.

Antibody	Clone	Type	Dilution
ER	SP1	rm	Ready to use
PgR	iE2	rm	Ready to use
Desmin	D33	mm	1:100
*α*SMA	1A4	mm	1:100
CD34	NU-4A1	mm	1:100
S-100 protein		rp	1:500
Ki-67	MIB-1	mm	1:100

mm: mouse monoclonal.

rm: rabbit monoclonal.

rp: rabbit polyclonal.

**(a):** 

	Histological features
Superficial myofibroblastoma (SMF)	Usually moderate, sometimes hypocellularity Fine and dense collagen in stroma Small to medium-sized dilated vessels, often centrally located

Cellular angiofibroma,	Moderate cellularity Wispy collagen stroma Medium-sized vessels, often with hyalinized walls

Mammary type myofibroblastoma,	Moderate to hypercellularity Thick collagen bundle in stroma, adipose tissues may be present Blood vessels are not prominent

Angiomyofibroblastoma, (AMF)	Alternating zones of hyper- and hypocellularity. Loose texture, contains mast cells in stroma, adipose tissues may be present Small to medium-sized capillary-like vessels around which epithelioid stroma cells are clustered

Aggressive angiomyxoma (AA)	Hypocellularity Myxoid stroma, entrapped tissue may be present Numerous blood vessels varying from small size to large size with thick muscular walls

**(b):** 

	ER	PgR	Desmin	SMA	CD34
Superficial myofibroblastoma	80-100% (+)	80-100% (+)	75-100% (+)	0-45% (+)	50-85% (+)

Cellular angiofibroma	variable expression	variable expression	variable expression	variable expression	variable expression

Mammary-type myofibroblastoma	ND	ND	usually expressed	variable expression	usually expressed

Angiomyofibro -blastoma	usually expressed	usually expressed	usually expressed	less common	less common

Aggressive angiomyxoma	(+)	(+)	(+)	variable expression	variable expression

## List of Abbreviations

SMF: Superficial myofibroblastoma
AA: Aggressive angiomyxoma
MRI: Magnetic resonance imaging.
